# Computed Tomography Evaluation of Extracellular Volume for Predicting Prognosis in Patients with Severe Renal Dysfunction on Dialysis

**DOI:** 10.3390/jcm13247561

**Published:** 2024-12-12

**Authors:** Hiroki Goto, Hiroyuki Takaoka, Joji Ota, Yoshitada Noguchi, Yusei Nishikawa, Moe Matsumoto, Kazuki Yoshida, Katsuya Suzuki, Shuhei Aoki, Satomi Yashima, Makiko Kinoshita, Haruka Sasaki, Noriko Suzuki-Eguchi, Yoshio Kobayashi

**Affiliations:** 1 Department of Cardiovascular Medicine, Chiba University, Chiba 260-8677, Japan; 2 Department of Radiology, Chiba University Hospital, Chiba 260-8677, Japan

**Keywords:** extracellular volume, dialysis, computed tomography, myocardial fibrosis

## Abstract

**Introduction**: Extracellular volume (ECV) analysis using computed tomography is recognized as a potential method for diagnostic application. It is currently the only noninvasive method for quantitatively evaluating myocardial fibrosis in dialysis patients for whom gadolinium contrast agents are contraindicated. In this study, we assessed the utility of ECV measurement via CT in the left ventricular (LV) myocardium (LVM) to predict major adverse cardiac events (MACEs) in dialysis patients. **Materials and methods**: We analyzed 57 dialysis patients who underwent cardiac CT and assessed the utility of LVM ECV (LV-ECV) for predicting MACEs. All the patients were followed for a median of 11 months, and MACEs occurred in 15 cases (26%). **Results**: LV-ECV and plasma brain natriuretic peptide levels were higher in subjects with MACEs than those without (40.29 ± 8.23% vs. 33.76 ± 4.60% and 1481 ± 997 vs. 807 ± 1109 pg/mL; both *p* < 0.05). Significant valvular disease was more frequently detected in patients with MACEs than those without (60% vs. 24%; *p* = 0.023). Serum hematocrit levels were significantly lower in patients with MACEs than those without (29 ± 5 vs. 34 ± 5; *p* < 0.001). The administration of statin was significantly lower in patients with MACEs than in those without (13% vs. 48%; *p* = 0.029). A receiver operating characteristic (ROC) curve analysis was performed using LV-ECV for predicting MACEs. The area under the curve was 0.80, and the best cut-off value of LV-ECV was 37.26% (*p* = 0.0003). In a Cox proportional hazards model, LV-ECV ≥ 37.26% was the only significant independent predictor of MACEs (*p* = 0.020). **Conclusions**: LV-ECV measured using CT is a useful predictor of MACEs in dialysis patients.

## 1. Introduction

Cardiorenal syndrome is commonly encountered in daily clinical practice. Cardiorenal syndrome (CRS) refers to a spectrum of clinical disorders in which acute or chronic dysfunction of the heart and kidneys is interrelated such that the failure of one organ contributes to or exacerbates the dysfunction of another. Chronic kidney disease (CKD) is accompanied by chronic heart failure (CHF) as a comorbidity in 26% to 63% of patients [1].

Cardiorenal syndrome (CRS) type 5 refers to a condition in which both the heart and kidneys are affected due to a systemic illness or disease. In this type of CRS, a primary systemic condition, such as sepsis, diabetes, systemic lupus erythematosus (SLE), or amyloidosis, causes simultaneous dysfunction in both the heart and the kidneys. Unlike the other types of CRS, where the dysfunction of one organ leads to the impairment of the other (heart–kidney or kidney–heart), CRS type 5 involves a third factor or illness that independently impacts both organ systems. Systemic inflammation, endothelial dysfunction, and neurohormonal dysregulation are common mechanisms that contribute to this dual organ involvement in CRS type 5. The management of CRS type 5 usually focuses on treating the underlying systemic condition to improve both cardiac and renal function.

Patients with severe renal dysfunction experience myocardial damage for various reasons, and the reference standard to evaluate this damage is the analysis of late enhancement (LE) using magnetic resonance imaging (MRI) [2]. Recently, extracellular volume (ECV) analysis using MRI has emerged as an alternative. ECV is a noninvasive imaging parameter used to quantify the proportion of the myocardium composed of extracellular space, where fluid and other materials exist outside of cells. The ECV provides significant prognostic information in cases of dilated cardiomyopathy (DCM), showing that patients with adverse cardiac events have a significantly higher ECV [3]. The ECV is also known as the best predictor of the risk of sudden cardiac death (SCD), with an area under the curve (AUC) of 0.83 in cases of hypertrophic cardiomyopathy (HCM) [4]. This is because a high ECV is associated with greater myocardial damage, which may lead to low cardiac function and arrhythmic adverse cardiac events. However, the utility of the ECV in patients on dialysis to predict future major adverse cardiac events (MACEs) is unknown.

Nephrogenic systemic fibrosis (NSF) is a potential complication of gadolinium-based contrast media used in MRI [5]. While the skin is most commonly affected, NSF can also involve other organs, such as the lungs, heart, diaphragm, and liver, and may lead to death. Due to the risk of NSF, gadolinium contrast agents are contraindicated in patients with severe renal dysfunction. Cardiac computed tomography (CT) plays a crucial role in detecting coronary artery stenosis in patients suspected of having myocardial abnormalities [6]. Additional late-phase contrast scans are valuable in detecting myocardial damage via myocardial LE [7]. Recent advancements in imaging software have also enabled ECV analysis in CT, with ECV values in CT showing a strong correlation with those obtained from MRI [8]. However, the predictive value of ECV in patients on dialysis for future prognosis has not been evaluated to date.

This study aimed to assess the usefulness of ECV in CT to predict prognosis in cases of severe renal dysfunction during dialysis.

## 2. Materials and Methods

This retrospective study included 60 consecutive adult patients (both males and females) on dialysis who were suspected of having coronary artery stenosis and underwent coronary computed tomography (CT) in our institution between December 2008 and May 2022. All patients on dialysis underwent transthoracic echocardiography (TTE) and cardiac CT, including late-phase imaging at the same tube voltage in the non-contrast phase. We excluded patients with cardiomyopathies or congenital heart diseases (one hypertrophic cardiomyopathy, one amyloidosis, and one coronary pulmonary artery fistula patient) from this analysis because these conditions could be prognostic determinants. Finally, the remaining 57 consecutive patients were analyzed. A composite of all-cause deaths and hospitalizations due to heart failure were included in MACEs. All patients were recommended a diet for renal impairment. Patient background and prognosis were recorded in the patient information in our institution. Patients were treated according to the guidelines in force at the time of treatment. The procedures followed the “Declaration of Helsinki” and the ethical standards of the responsible committee on human experimentation. The Ethics Committee of the Chiba University Graduate School of Medicine approved this retrospective study and granted a waiver for informed consent (reference number 3822).

### 2.1. Protocol for Computed Tomography

The CT protocol we used has previously been published in [7], with modifications to some key points. CT was performed using a 320-row detector CT (Aquilion One or Aquilion One/ViSION Edition, Canon Medical Systems, Otawara, Japan) or a 256-row detector CT (Revolution APEX, GE Healthcare, Waukesha, WI, USA).

#### 2.1.1. Non-Contrast Scan

Scout and non-contrast prospective ECG-gated cardiac scans were performed before contrast scans. CT was performed with a slice thickness of 0.5 mm and a tube voltage of 80–120 kV for the 320-row detector CT or a slice thickness of 0.625 mm and tube voltage of 70 kV for the 256-row detector CT. The tube current for non-contrast scanning was determined via an automatic exposure control (AEC) system.

#### 2.1.2. Post-Contrast Scan (Early Phase)

Retrospective ECG gating with dose modulation was utilized for early-phase post-contrast scans, where applicable. Conventional contrast-enhanced CT was performed with a slice thickness of 0.5 mm and a tube voltage of 80–120 kV for the 320-row detector CT or a slice thickness of 0.625 mm and a tube voltage of 120 kV for the 256-row detector CT [7,9]. The tube current was determined via automatic exposure control (AEC). All patients with a heart rate above 65 beats per minute were administered 10 mg of metoprolol or 12.5 mg of landiolol immediately before the scan, except those for whom β-blockers were contraindicated. Landiolol was introduced midway through this study; therefore, metoprolol was used in the earlier cases and landiolol in the later cases. Additionally, two doses of sublingual isosorbide dinitrate were given to dilate the coronary artery just before the scan.

The contrast agent was injected using a standard triphasic protocol to ensure clear visualization of the right coronary artery with high contrast and moderate contrast for the right ventricle and atrium. Intravenous access was obtained via the right or left antecubital vein using a 20- or 22-gauge needle, and the system was connected to a dual-syringe injector with a dual-flow function (Dual Shot, Nemoto, Tokyo, Japan). In the first phase, 50–100 mL of an undiluted iodinated contrast agent (350–370 mg/mL) was injected at 3–5 mL/s, followed by 40–50 mL of a 50% saline-to-contrast mixture at 3–4 mL/s, and then 20–30 mL of pure saline at 2–4 mL/s. Time-resolved (1 s intervals) single-section CT scans were acquired at the mid-left ventricle level, starting 10 s after contrast injection and continuing until approximately 40 s. The actual scan was performed while the patient held their breath once the CT values in the descending aorta had reached 200 HU [7].

#### 2.1.3. Post-Contrast Scan (Late Phase)

A prospective ECG-gating scan was conducted 6 min after the administration of an iodine contrast agent as a late-phase scan [7,10]. The slice thickness and tube voltage matched those used for the non-contrast scans. The tube current for the late-phase scan was set using automatic exposure control (AEC).

### 2.2. Analysis of ECV in CT

**Figure 1:**
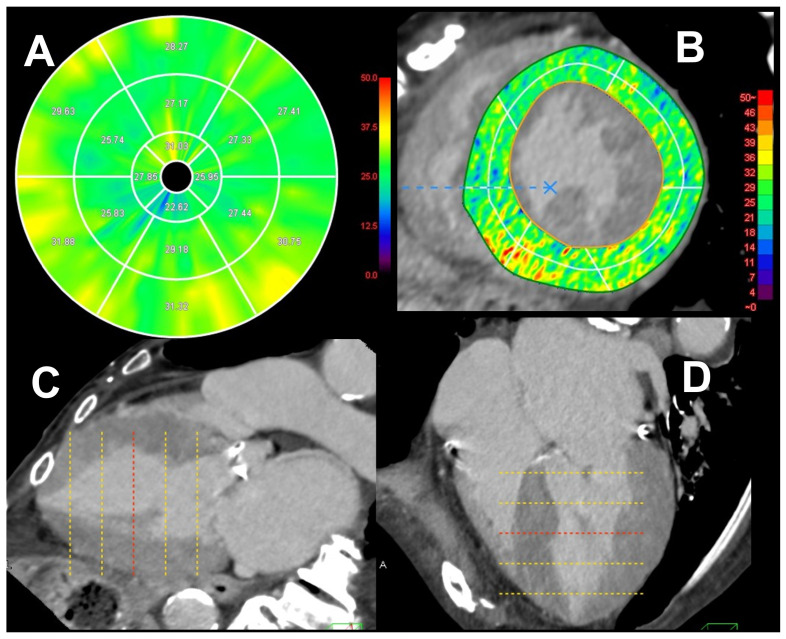
Details for left ventricular extracellular volume analysis in CT. The left ventricular (LV) extracellular volume (ECV) (LV-ECV) measurements were obtained from non-contrast and late-phase cardiac CT scans using commercially available software (Ziostation 2, Ziosoft Inc, Tokyo, Japan). Segmental LV-ECV was analyzed based on the 16-segment model of the LVM (**A**). ECV analysis was performed using short-axis images and two- and four-chamber views of the late-phase cardiac CT images (**B**–**D**). The yellow dotted line in the LV long-axis image shows the short-axis slice for analysis, while the image presented in the top right corner shows the cut plane of the red dotted line.

A commercially available software product (Ziostation 2, Ziosoft Inc., Tokyo, Japan) was used for ECV analysis of the left ventricular (LV) myocardium (LVM). The ECV was calculated using the following formula: ECV = (ΔHUm/ΔHUb)/(1 − Hematocrit), where ΔHUm indicates the change in myocardial CT attenuation in Hounsfield units (HU), and ΔHUb indicates the change in the CT attenuation of the blood [8,11,12] (Figure 1).

First, subtraction images were generated by combining non-contrast and late-phase cardiac CT images. The LV-ECV was calculated using the difference in CT attenuation between the LV lumen and myocardium. The LV-ECV measurements were obtained from non-contrast and late-phase cardiac CT scans using commercially available software. Segmental LV-ECV was analyzed based on the 16-segment model of the LVM, and the average LV-ECV values were displayed on a polar map (Figure 1A). ECV analysis was performed using short-axis images and two- and four-chamber views of the late-phase cardiac CT images (Figure 1B–D).

The software enabled the automatic three-dimensional non-rigid registration of the myocardium between non-contrast and late-phase CT images, allowing for the creation of subtraction images [7,10,11]. CT attenuation changes (ΔHU) were measured in these subtraction images. A polar map was then generated, illustrating the mean ECV values for each of the 16 American Heart Association myocardial segments and the overall mean ECV value for the entire LVM (Figure 1A) [7,12].

For the ECV analysis, a cardiologist (H.G.) with four years of experience in cardiac CT measured the LV-ECV values, which were included in subsequent analyses. An additional cardiologist (H.T.) with 14 years of experience in cardiac CT independently measured the LV-ECV values, and the inter-observer agreement was assessed. Cases with significant coronary artery stenosis (>70% or >50% in the left main coronary artery) were analyzed by both observers using the 15-segment American Heart Association model, excluding small branches with diameters of <1.5 mm [13]. Any discrepancies regarding significant coronary stenosis were resolved by consensus. Visible LE of the LVM was assessed using the 17-segment model (evaluation by H.T.) [12]. The contrast-to-noise ratio (CNR) was also evaluated for cases with visually assessed LE in CT.

**Figure 2:**
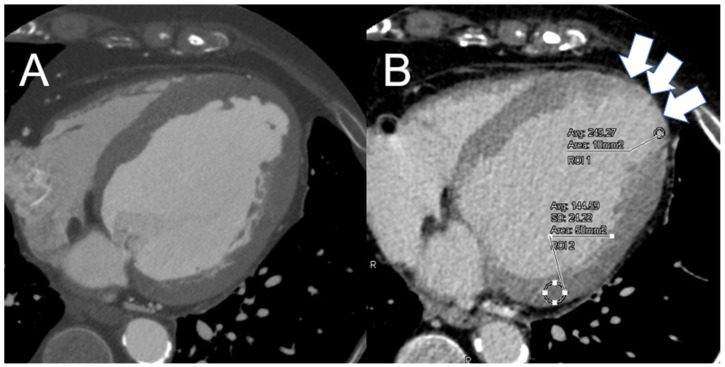
Quantitative image quality analysis. (**A**) Axial early contrast phase cardiac computed tomography image; (**B**) Axial late contrast phase cardiac computed tomography image in the same case as (**A**). Quantitative image quality analysis was conducted by placing regions of interest (ROIs) measuring approximately 10 mm^2^ in the LE regions and 50 mm^2^ in the remote myocardium without late enhancement (LE). The ROIs were manually selected in the LE areas (white arrows in (**B**), ROI 1) and the remote normal myocardium (ROI 2 in (**B**)). Computed tomography (CT) attenuation values were measured in patients showing LE in the LVM in CT. The contrast-to-noise ratio (CNR) was calculated as the difference in attenuation between the LE and remote normal myocardium, divided by the standard deviation (SD) of the attenuation in the remote normal myocardium.

Figure 2A,B illustrate axial early- and late-contrast-phase cardiac CT images from a patient with an old LV apical myocardial infarction, demonstrating the method used for CNR evaluation.

Quantitative image quality analysis was conducted by placing regions of interest (ROIs) measuring approximately 10 mm^2^ in the LE regions and 50 mm^2^ in the remote myocardium without LE. The ROIs were manually selected in the LE areas and the remote normal myocardium. CT attenuation values were measured in patients showing LE in the LVM in CT (Figure 2B). The contrast-to-noise ratio (CNR) was calculated as the difference in attenuation between the LE and remote normal myocardium, divided by the standard deviation (SD) of the attenuation in the remote normal myocardium [10,14]. Additionally, the coronary artery calcium score, expressed as the Agatston score, was assessed by a single observer (H.T.) using the same software.

### 2.3. Statistical Analysis

Continuous variables are reported as means ± standard deviations (SDs), and categorical variables are presented as frequencies and percentages. All tests were two-sided, with a *p*-value of <0.05 considered to indicate statistical significance. Comparisons of continuous variables were conducted using Student’s *t*-test. Fisher’s exact test was applied for categorical variables. Pearson’s correlation coefficient was used to evaluate the agreement between two observers’ LV-ECV measurements in CT.

A receiver operating characteristic (ROC) analysis was performed to predict MACEs, with the optimal cut-off value identified using the Youden index. A Kaplan–Meier analysis was utilized to estimate time to MACE and calculate MACE rates using the log-rank test for comparisons between groups. A Cox proportional hazards model was also employed to examine the relationships between predictor variables and time to MACE. JMP Pro version 15 software (SAS Institute Inc., Cary, NC, USA) was used for all statistical analyses.

## 3. Results

Fifty-seven consecutive patients (69 ± 12 years old) were followed for a median of 11 months after cardiac CT. A total of 15 patients (26%) had MACEs during the follow-up period. Five patients were admitted to the hospital because of congestive heart failure. Another five patients died from cardiovascular diseases. Another three patients died because of non-cardiac diseases, and the remaining two patients died for unknown reasons. Patient background, including risk factors of coronary artery disease, treatments, and CT or TTE findings, were compared between those with and without MACEs, as shown in Table 1.

There was no significant difference in age, gender difference, or past history between the patients with and without MACEs. The patients with MACEs had a higher percentage of significant valvular disease (at least moderate on TTE) than did those without. Additionally, they had comparatively higher serum BNP levels and ECV in CT. Conversely, they had lower hematocrit levels and statin use.

**Figure 3:**
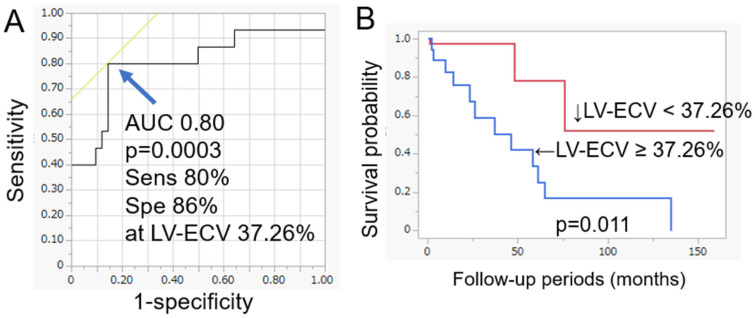
Receiver operating characteristic curve (**A**) and Kaplan–Meier survival curves to predict major adverse cardiac events (**B**).

The area under the receiver operating characteristic curve was 0.80, and the best cut-off value of LV-ECV was 37.26% (Figure 3) (*p* = 0.0003).

The area under the receiver operating characteristic curve was 0.80, and the best cut-off value of left ventricular (LV) extracellular volume (ECV) (LV-ECV) was 37.26% (Figure 3) (*p* = 0.0003). The sensitivity and specificity for the prediction of major adverse cardiac events (MACEs) at the best cut-off value of LV-ECV in computed tomography (CT) were 80% and 81% (*p* = 0.0003) (Figure 3A). Based on the Kaplan–Meier analysis, cases with ECV ≥ 37.26% had a significantly more frequent occurrence of MACEs during the follow-up period than those without (*p* = 0.011) (Figure 3B).

The sensitivity and specificity for the prediction of MACEs at the best cut-off value of LV-ECV in CT were 80% and 81% (*p* = 0.0003) (Figure 3A). Based on the Kaplan–Meier analysis, cases with ECV ≥ 37.26% had a significantly more frequent occurrence of MACEs during the follow-up period than those without (*p* = 0.0112) (Figure 3B). The background data and CT and TTE findings were also compared between the patients with LV-ECV ≥ 37.26% and those without. The percentage of significant valvular disease was significantly higher in patients with LV-ECV ≥ 37.26% (61% vs. 21%; *p* = 0.0054) (Table 2).

Additionally, serum hematocrit values were significantly lower (30 ± 5% vs. 34 ± 5%; *p* = 0.0188), while serum BNP was significantly higher (1485 ± 1038 vs. 740 ± 1085 [mg/dL]; *p* = 0.0207), in cases with LV-ECV ≥ 37.26% than those without.

In a univariate and multivariate Cox proportional hazards model, only LV-ECV ≥ 37.26% in CT was a significant independent predictor of MACE during the follow-up period (*p* = 0.020) (Table 3).

The CNR of LE of LVM in CT was 3.6 ± 1.6. Pearson’s correlation coefficient used to assess the consistency between the two observers’ evaluations of LV-ECV in CT was 0.85. The CT dose index for the late-phase scans was 13.3 ± 5.8 mGy, as recorded for all patients who underwent cardiac CT except for the first five, for whom radiation dose details were missing from the reporting system.

## 4. Discussion

In this study, we found that LV-ECV in CT may be a predictor of future events in patients on dialysis. This study’s main purpose was to predict the prognosis of dialysis patients. We considered that cardiac function, the presence or absence of valvular disease, BNP levels, and the presence or absence of cardioprotective drugs could be confounding factors. However, after conducting a proportional hazards analysis, only the ECV results in CT emerged as a significant prognostic factor. CT is useful for detecting coronary artery stenosis, and ECV analysis is also possible with additional late-phase scanning. The ECV values of LVM in CT correlated well with those in MRI in previous research [8]. Patients on dialysis have numerous risk factors for coronary artery disease and are subject to hemodynamic changes induced by atrioventricular shunt and periodic dialysis, so it is important to screen for coronary artery disease and cardiac function in these patients. However, gadolinium contrast agents are contraindicated because of the risk of NSF, and CT is the only suitable method available for evaluating myocardial damage in cases of abnormal cardiac function [5]. CT is, accordingly, a highly useful modality for whole cardiac screening in dialysis patients requiring risk assessment. However, ECV in CT has not previously been assessed and compared with other risk factors, and it is a potential new and significant risk stratification factor for patients on dialysis. We did not perform multivariable analysis because some parameters are correlated with LV-ECV, which may affect the analysis results.

### 4.1. ECV Analysis in Patients on Dialysis

ECV analysis using CT is the only alternative to MRI with gadolinium contrast agents for patients on dialysis. Yamada et al. [11] investigated ECV analysis using CT and its utility in dialysis cases, in which the reported LV-ECV was higher than that in the controls. Myocardial damage occurs in cases of severe renal dysfunction for a variety of reasons, including anemia, malnutrition, and chronic inflammation. Renal dysfunction ultimately results in cardiac remodeling, myocardial hypertrophy, and tissue calcification in the heart and accelerates sclerosis, endothelial dysfunction, smooth muscle cell proliferation, and vessel calcification [1]. Renal dysfunction is also caused or accelerated by cardiac disease because of lower cardiac output, the several adverse effects of medicines used for heart failure, increased venous pressure, and chronic activated inflammation [1]. Therefore, detecting background cardiac disease in these patients is important for the risk assessment of cardiac events during dialysis.

Although an additional radiation dose is required for ECV analysis in CT, this is considered an acceptable tradeoff for patients on dialysis, given the important clinical information that is obtained, such as the ECV. The CT dose index for late-phase scans was 13.3 ± 5.8 mGy in this study. As a result of new iterative reconstruction and wide-coverage CTs, the dose needed to obtain late-phase cardiac images with improved image quality has recently decreased [15]. Together, these factors have ameliorated the disadvantage of additional late-phase cardiac imaging, which should be recommended for patients on dialysis.

### 4.2. Relationship Between MACE and ECV

Numerous previously identified risk factors for patients on dialysis, including male sex, age, the presence of diabetes, a history of cardiovascular disease, the cardiothoracic ratio, serum CRP levels, and serum phosphate levels, were recently demonstrated to be independent predictors of sudden death [16]. In our present study, however, these risk factors were not significant. Only LV-ECV measured in CT was shown to be a significant risk factor for events.

Increased LV-ECV is closely correlated with higher amounts of biopsy-proven myocardial fibrosis [17,18]. Therefore, a higher ECV indicates severe degeneration of the LVM, which, in turn, indicates an increased risk of decreased LV function or ventricular arrhythmic events [4]. Based on our results, dialysis patients with higher LV-ECV in CT tended to have significant valvular disease or higher plasma BNP levels. Therefore, CT appears to be useful for detecting myocardial damage caused by valvular abnormalities, and LV-ECV in CT correlates well with higher plasma BNP levels. The degree of myocardial fibrosis is a known significant risk factor for future cardiac events in several myocardial conditions, and our findings align with this association [3,4,7,19].

ECV analysis typically requires contrast media, and there is a risk of residual renal function in cases where it has not been abolished. Initially, ECV analysis was only performed using MRI, but MRI contrast agents are contraindicated for dialysis patients and, therefore, not feasible in such cases. Recently, a new software package has enabled ECV analysis with CT; it is a useful alternative for predicting patient prognosis. ECV analysis can be performed by only adding the late phase in cases of cardiac CT, which has the advantage of not incurring additional costs. The image quality of CT is often inferior in the late contrast-enhanced phase compared with MRI, and there is a risk that motion artifacts may lead to overestimation of the ECV, resulting in inaccurate interpretations of patients’ results.

### 4.3. Improving Image Quality in Late-Phase Enhancement

The contrast resolution of late-phase cardiac CT images is lower than that of MRI, which remains the gold standard for evaluating myocardial damage in late-phase imaging. While reducing the tube voltage during CT scans can enhance attenuation values, it also increases image noise due to radiation dose limitations [20]. Recently, iterative and deep learning reconstruction techniques have emerged as promising alternatives, alongside an increase in the maximum tube currents, both of which help mitigate image noise at lower tube voltages. Furthermore, dual-energy acquisition and dual-layer CT have proven effective in improving the quality of LE images in CT. Our previous research demonstrated that combining next-generation CT technology with iterative reconstruction techniques enhances the quality of LE imaging, improving the diagnostic accuracy for detecting myocardial fibrosis [15]. These advancements have contributed to the inclusion of CT-based ECV analysis for cardiac amyloidosis in the latest guidelines by the Japanese Circulation Society [21].

LE in the LVM is a recognized risk factor in patients with specific cardiomyopathies [18]. However, in this study, the incidence of LVM LE did not significantly differ between patients with and without MACE. One possible explanation is CT’s limited sensitivity in detecting LVM LE. Nevertheless, a contrast-to-noise ratio (CNR) of approximately four was achieved in 22 patients (39%) with LVM LE in this study. In cases of old myocardial infarction, previous studies reported CNR values for late-phase CT scans ranging from nearly six in one study to two in another. Despite differences in the underlying myocardial conditions, the CNR values observed in this study were comparable to those reported in prior research involving patients with old myocardial infarction [10,14].

### 4.4. Clinical Significance of This Study

There are no previous reports on the utility of ECV in CT for predicting prognosis in patients on dialysis. Additionally, ECV analysis was originally performed using MRI, which is contraindicated for dialysis patients due to the use of MRI contrast media. Therefore, this study is significant because it demonstrates that CT alone can be used to predict the prognosis of dialysis patients, which is usually poor.

Additionally, ECV analysis with CT is easy to perform because, aside from additional imaging of the contrast-enhanced late phase, it can be performed in only a few minutes using commercially available image analysis software.

Considering the inherently high risk of atherosclerosis in dialysis patients, when cardiac CT is necessary, contrast-enhanced late-phase and ECV analysis should be actively utilized in addition to coronary artery stenosis assessment. In cases with high values, further evaluation of myocardial damage should be performed using myocardial biopsy, and the aggressive addition of cardioprotective drugs should be considered.

### 4.5. Limitations

This study has several limitations. First, it was performed at a single center under a retrospective design, and the results might only apply to dialysis patients with suspected coronary artery disease who are candidates for cardiac CT. Therefore, confirmation of the results requires a larger prospective study. Second, ECV analysis was performed on single-energy images, therefore requiring the subtraction of late-phase and non-contrast images. Gaps between these images might have caused ECV under- or overestimation in the analysis of single-energy images compared with dual-energy images (without gaps). Thirdly, three types of CT scanners were applied in this study population. Therefore, the difference in the performance of each scanner might have affected the results. In particular, the image quality of LE of LVM in CT might have been lower for patients who underwent cardiac CT at an early stage than those who underwent CT later. Fourth, it was difficult to adequately monitor nutritional status, quality of life, and patient compliance, which may have impacted the findings.

## 5. Conclusions

Evaluating ECV using CT is useful for predicting prognosis in patients with renal dysfunction on dialysis.

## Figures and Tables

**Table 1:** Comparison of patient background, treatments, and CT or TTE findings between patients with and without MACEs.

		MACEs (+) (N = 15)	MACEs (-) (N = 42)	*p*-Value
Age, years		66 ± 9	70 ± 13	0.293
Male, n (%)		11 (73)	29 (69)	1.000
Body mass index, kg/m^2^		23 ± 4	23 ± 4	0.789
Past history	Hypertension, n (%)	13 (87)	30 (71)	0.312
	Dyslipidemia, n (%)	7 (47)	18 (43)	1.000
	Diabetes mellitus, n (%)	6 (40)	17 (40)	1.000
	Atrial fibrillation, n (%)	2 (13)	5 (12)	1.000
	Post-mechanical device implantation, n (%)	3 (20)	5 (12)	0.422
	Post-coronary artery revascularization, n (%)	4 (27)	17 (40)	0.534
	Post-valvular surgery, n (%)	3 (20)	4 (10)	0.365
	Old myocardial infarction (%)	6 (40)	8 (19)	0.161
Familial history of coronary artery disease, n (%)		0 (0)	5 (12)	0.314
Hematocrit (%)		29 ± 5	34 ± 5	<0.001
Serum phosphorus level (mg/dL)		4.1 ± 1.9	4.7 ± 1.4	0.186
Serum CRP level (mg/dL)		3.8 ± 5.2	1.7 ± 3.2	0.075
Plasma BNP level (pg/dL)		1481 ± 997	807 ± 1109	0.0499
CTR in chest X-ray (%)		57.7 ± 5.0	55.9 ± 7.9	0.420
TTE findings	LVEF in TTE (%)	50 ± 17	54 ± 13	0.392
	Average E/e′ of LVM in TTE (%)	14 ± 4	16 ± 9	0.331
	Significant valvular disease (≥moderate) in TTE, n (%)	9 (60%)	10 (24%)	0.023
	Left atrial volume (mL)	66 ± 22	72 ± 36	0.572
Cardiac CT findings	Significant coronary artery stenosis in CT, n (%)	8 (53%)	26 (62%)	0.760
	Agatston calcium score	1371 ± 1234	3457 ± 8327	0.341
	Presence of late enhancement of LVM in CT	3 (20%)	19 (45%)	0.124
	ECV of LVM in CT	40.29 ± 8.23	33.76 ± 4.60	0.0004
Treatments	Revascularization of coronary arteries during follow-up	5 (33)	10 (24)	0.507
	Valvular surgery during follow-up	3 (20)	8 (19)	1.000
	Administration of β-blocker, n (%)	7 (47)	19 (45)	1.000
	Administration of statin, n (%)	2 (13)	20 (48)	0.029
	Administration of ACE-i or ARB, n (%)	6 (40)	17 (40)	1.000

Serum phosphate levels were not measured in four cases, and CTR in chest X-ray and serum CRP levels were not measured in one case. Plasma BNP levels and average E/e′ of LVM were not measured in three cases. Left atrial volume was not measured in four cases. ACE, angiotensin-converting enzyme; ARB, angiotensin receptor II blocker; CRP, C-reactive protein; CTR, cardiothoracic ratio; BNP, brain natriuretic peptide; LV, left ventricle; TTE, transthoracic echocardiography; LVM, left ventricular myocardium; CT, computed tomography.

**Table 2:** Comparison of patient background between patients with and without left ventricular myocardium extracellular volume ≥ 37.26 (%).

		ECV ≥ 37.26 (%) (N = 18)	ECV < 37.26 (%) (N = 39)	*p*-Value
Age, years		65 ± 10	70 ± 13	0.180
Male, n (%)		15 (83%)	25 (64%)	0.214
Body mass index, kg/m^2^		23 ± 4	23 ± 4	0.881
Hematocrit (%)		30 ± 5	34 ± 5	0.019
Familial history of coronary artery disease, n (%)		0 (0%)	5 (13%)	0.310
Medical treatments	Administration of β-blocker, n (%)	8 (44%)	18 (46%)	1.000
	Administration of statin, n (%)	5 (28%)	17 (44%)	0.381
	Administration of ACE-i or ARB, n (%)	7 (39%)	16 (41%)	1.000
	Revascularization of coronary arteries during follow-up	4 (22%)	10 (28%)	1.000
	Valvular surgery during follow-up	3 (17%)	8 (21%)	1.000
Serum phosphorus level (mg/dL)		4.1 ± 1.6	4.8 ± 1.5	0.103
Serum CRP level (mg/dL)		3.3 ± 4.9	1.8 ± 3.2	0.147
Plasma BNP level (pg/dL)		1485 ± 1038	750 ± 1080	0.023
CTR in chest X-ray (%)		59.1 ± 6.2	55.1 ± 7.4	0.050
Past history	Hypertension, n (%)	13 (72%)	30 (77%)	0.702
	Dyslipidemia, n (%)	6 (33%)	19 (49%)	0.391
	Diabetes mellitus, n (%)	8 (44%)	15 (38%)	0.669
	Atrial fibrillation, n (%)	4 (22%)	3 (8%)	0.191
	Post-coronary artery revascularization, n (%)	7 (39%)	15 (38%)	1.000
	Post-valvular surgery, n (%)	4 (22%)	3 (8%)	0.191
	Old myocardial infarction (%)	6 (33%)	8 (21%)	0.334
TTE findings	LVEF in TTE (%)	50 ± 15	54 ± 13	0.329
	Average E/e’ of LVM on TTE (%)	14 ± 5	16 ± 10	0.430
	Significant valvular disease (≥moderate) in TTE, n (%)	11 (61%)	8 (21%)	0.005
	Left atrial volume (mL)	74 ± 23	69 ± 37	0.551
CT findings	Significant coronary artery stenosis in CT, n (%)	10 (56%)	24 (62%)	0.774
	Agatston calcium score	1912 ± 2077	3367 ± 8064	0.484

Serum phosphate levels were not measured in four cases, and CTR in chest X-ray and serum CRP levels were not measured in one case. Plasma BNP levels and average E/e’ of LVM were not measured in three cases. Left atrial volume was not measured in four cases. ACE, angiotensin-converting enzyme; ARB, angiotensin receptor II blocker; CRP, C-reactive protein; CTR, cardiothoracic ratio; BNP, B-type natriuretic peptide; TTE, transthoracic echocardiography; LV, left ventricle; LVM, left ventricular myocardium.

**Table 3:** Cox proportional hazards model of risk factors for major adverse cardiac events.

	Univariate	
	Hazard Ratio (95% Confidence Interval)	*p*-Value
Hematocrit (%)	0.933 (0.831–1.041)	0.218
Plasma BNP level (pg/dL)	1.000 (0.9999–1.001)	0.105
LV-ECV in CT ≥ 37.26 (%)	4.585 (1.28–16.48)	0.020
Significant valvular disease (≥moderate) in TTE, n (%)	2.597 (0.918–7.351)	0.072
Administration of Statin	0.8212 (0.1655–6.041)	0.557

LV, left ventricle; ECV, extracellular volume; BNP, B-type natriuretic peptide; TTE, transthoracic echocardiography.

## Data Availability

No data related to this study will be shared.
